# The AO triangular external fixator: a backup option in the treatment of ankle fractures in geriatric patients?

**DOI:** 10.1007/s00590-020-02740-0

**Published:** 2020-11-06

**Authors:** Robert Hennings, Ulrich J. Spiegl, Johannes K. M. Fakler, Annette B. Ahrberg

**Affiliations:** Department of Orthopaedics, Traumatology and Plastic Surgery, University of Leipzig, Liebigstr. 20, 04103 Leipzig, Germany

**Keywords:** Ankle, Minimal-invasive, Geriatric, Fracture, External fixator

## Abstract

**Purpose:**

To analyze the indications, radiological short-term outcomes, and complications of ankle fractures in geriatric patients treated with a triangular external fixator (AEF) until fracture healing. Furthermore, the effect of an additional osteosynthesis to AEF on the radiological outcome was investigated.

**Methods:**

Retrospective analysis of ankle fractures treated in a Level I Trauma Center between 2005 and 2015 with an AEF in patients aged ≥ 65 years until fracture has healed. The combination of AEF and at least one additional osteosynthesis of a malleolus was defined as hybrid external fixator (HEF). At the time of AEF removal, a preserved ankle joint congruity was defined as good radiological outcome. Incongruity more than 2 mm was defined as poor radiologic results.

**Results:**

16 patients (13 women, 3 men) with a mean age of 74 years (SD 6.2) were treated with AEF until fracture healing, 9 with a single AEF and 7 with a HEF. Stabilization with HEF (*n* = 7 [100%]) showed higher rates of good radiological outcome than AEF alone (*n* = 4 [44%] of 9; *p *= 0.034). The duration of therapy did not differ between HEF and AEF (70 day vs 77 days). 4 patients (22%) required surgical revision.

**Conclusion:**

It could be shown that osteosynthesis in addition to AEF leads to a better radiological short-term results than using AEF alone. Therefore, in the situation where an AEF is considered as the definitive treatment option for an ankle fracture in geriatric patients with expected or existing soft tissue problems, it should be done or completed as a HEF.

**Level of evidence:**

Therapeutic level IV.

## Introduction

Ankle fractures are some of the most common fractures in geriatric patients and studies show that the incidence is increasing [1]. In geriatric patients, the quality of the soft tissue and bone is often reduced, comorbidities are increased and the fracture morphology after low-energy trauma is often more complex [2–4]. Therefore, complications related to, for example, wound closure, infections, or secondary dislocation occur at a higher rate than in younger patients [5]. Thus, the indications for surgical or conservative treatment of polymorbid elderly patients have been the subject of controversial discussions in the past. In recent literature, there is a consensus that the surgical treatment of highly unstable fractures leads to better outcome and lower mortality [6–8]. The primary aim of performing an operation is to ensure full load-bearing capability at the earliest time and maintaining the preinjury functional status [8, 9].

**Fig. 1:**
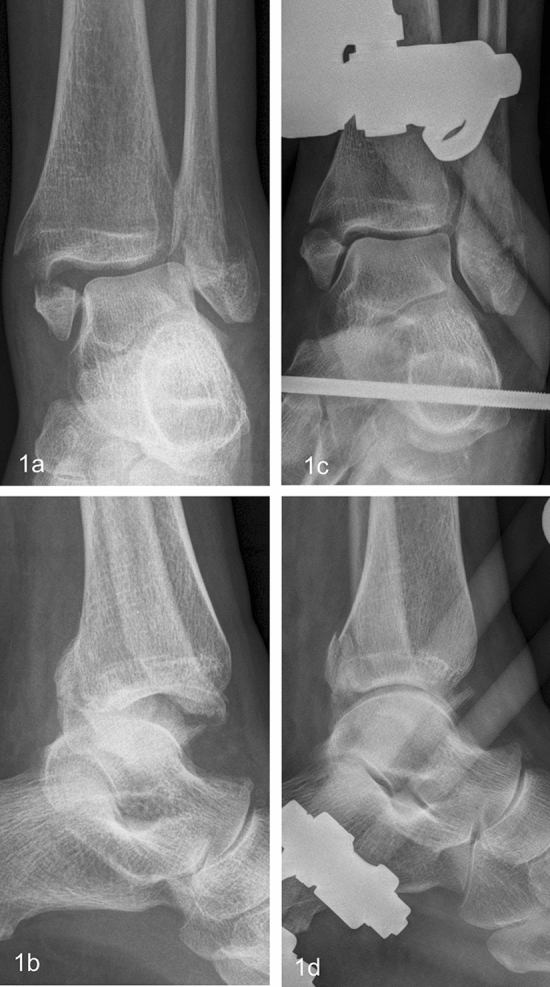
69-year-old female patient suffered an AO 44B3 fracture (**a**, **c**). Due to poor state of the soft tissue, the fracture was stabilized with an AEF on the day of trauma (**b**, **d**)

In emergency treatment, reduction and stabilization is recommended after immediate clinical examination with evaluation of the soft tissues, peripheral sensitivity and motor function in the event of dislocation [9–11]. Polyneuropathy, vulnerable skin with a tendency to blister, and the risk of infection represent contraindications to therapy in casts [11]. Furthermore, Meijer et al. [12] describe a secondary dislocation rate of up to 73% for primary cast stabilization. Therefore, closed reduction and stabilization with an external fixator (EF) is indicated in highly unstable fractures and/or relevant accompanying soft tissue damage that are not qualified for immediate open reduction and internal fixation (ORIF) or plaster stabilization (Fig. 1a–d) [10–13]. The Arbeitsgemeinschaft für Osteosynthesefragen (AO) triangular external fixator (AEF) is an ankle joint bridging fixator indicated for the purpose to enable the soft tissues to recover, thus reducing the complication rate of definitive treatment with open reduction and internal fixation (ORIF) with plate, which is the current gold standard [10–13].

Persistent poor soft tissue conditions as well as peritraumatic medical complications, such as cerebral or cardiac events despite EF, are associated with an increased risk of complications after ORIF. Therefore, after a case-by-case decision, techniques deviating from the gold standard should also be considered for definitive treatment [9, 12, 14]. To the authors’ knowledge, there are no short-term results of geriatric patients with poor soft tissue treated with the AO triangular external fixator (AEF) until fracture healing or whether an additive osteosynthesis has an impact on the radiological outcome. The aim of this study was to analyze the indications, complications, and radiographic short-term results of geriatric patients treated with AEFs as a definite treatment strategy. Furthermore, it should be investigated whether selective osteosynthesis in addition to AEF has an effect on the radiological outcome.

## Patients and materials

Institutional review board approval (488/19-ek) was given prior to data acquisition. Consecutive patients aged 65 years and older who suffered from an ankle fracture (AO 44) which were initially stabilized with an AO triangular external fixator (AEF) at a single Level I Trauma Center between 2005 and 2015 were retrospectively analyzed. The study period extended from hospital admission to the first radiological control after removal of EF, which was defined as the radiological short-term result and determined as the primary endpoint of the study.

All patients in whom AEF were determined as the treatment procedure until fracture healing for an AO 44 fracture were included for further analysis of preoperative mobility status, indications for EF, complications during therapy, and short-term radiographic outcome. Exclusion criteria were other initial fixator types, e.g., Ilizarov ring or double-frame fixator types because they were not generally used as first-line stabilization at the center, other stabilizations prior the EF and other diagnoses than fracture, e.g., infection or failed osteosynthesis.

**Fig. 2:**
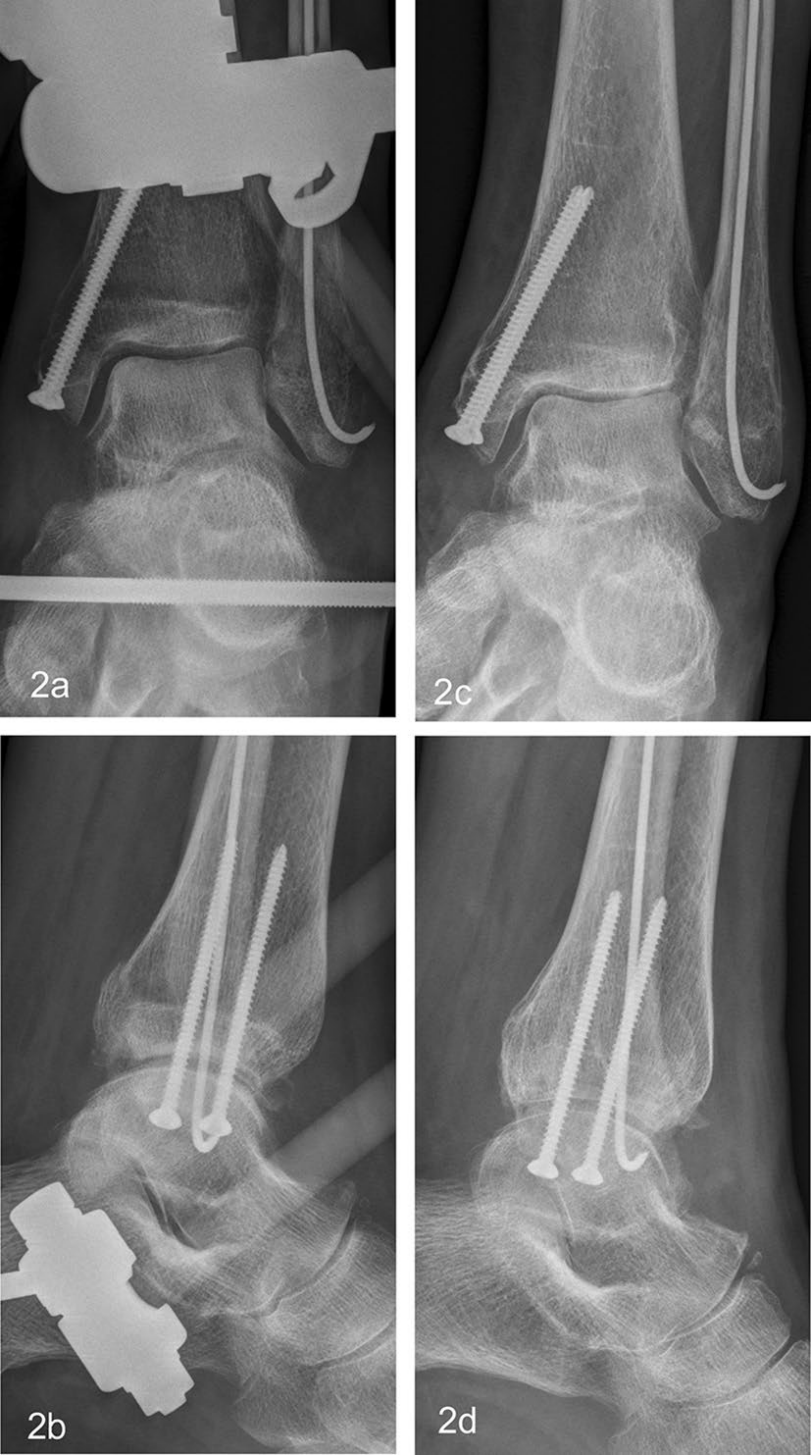
Same patient as in Fig. 1. Due to lack of recovery of the soft tissues, conversion with additive osteosynthesis into secondary HEF was performed 9 days after trauma (**a**, **c**). AEF removal 60 days after trauma with preserved congruity and joint space (**b**, **d**)

Fractures were classified according to the OTA/AO classification. All AEF corresponded to a pin and rod system and consisted of two percutaneously inserted Schanz-type pins at the anterior tibial crest, one Steinmann pin inserted horizontally through the calcaneal tuberosity, and two small pins in metatarsal 1 and 5 or 4, respectively. The frame is stabilized by carbon fiber rods. The combination of AEF and a simultaneous osteosynthesis of at least one malleolus (medial, lateral and/or posterior) was defined as AO hybrid external fixator (HEF; Fig. 2a–d). The osteosynthesis of the medial malleolus was done using either 3.5 mm cortical screws or Kirschner-(K-)wires, and the fibula was stabilized using cortical screws (DepuySynthes, Warsaw, IN, USA), k-wires between 1.6 and 2.5 mm (Königsee, Allendorf, Germany or B.Braun Aesculap, Tuttlingen, Germany), intramedullary elastic nails (Königsee, Allendorf, Germany), and one-third tubulare plates (DepuySynthes, Warsaw, IN, USA). To classify the patients’ preoperative physical status, the American Society of anesthesiologists (ASA) physical status was used, that is routinely documented during preoperative anesthesiological assessment [15]. In addition, the documented preoperative living conditions (self-employed or nursing home) were included. A low-energy trauma was defined as a fall from standing height while walking. The high-energy traumas included sport injuries, falls from stairs or car accidents [16]. All patients were mobilized at the hospital with partial weight bearing of 20 kg and orthopedic walking aids under the supervision of a physiotherapist.

**Fig. 3:**
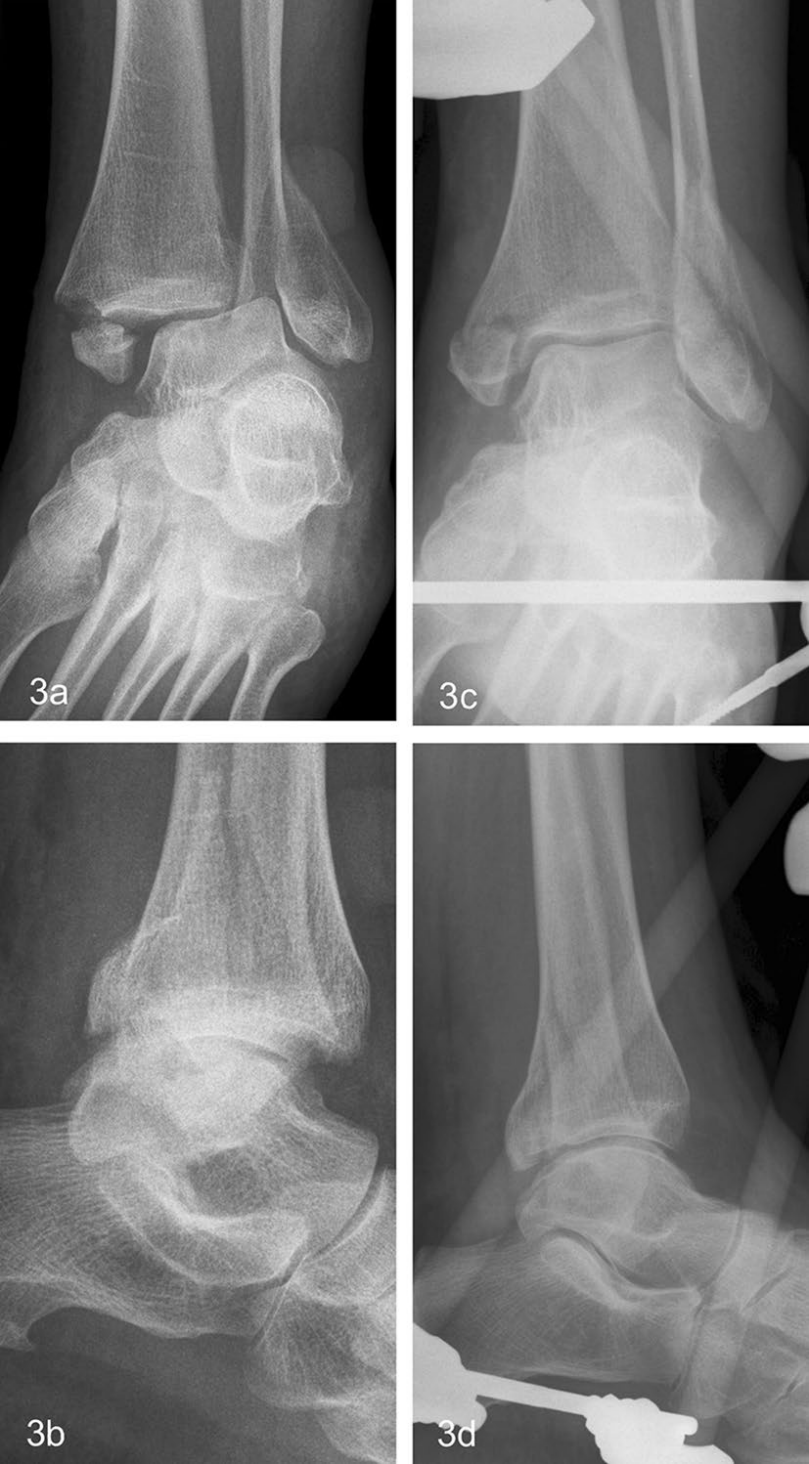
68-year-old female patient suffered an AO 44B3 fracture (**a**, **c**). Due to incongruity in the cast and poor soft tissues, stabilization with an AEF was done 3 days posttraumatic (**b**, **d**)

**Fig. 4:**
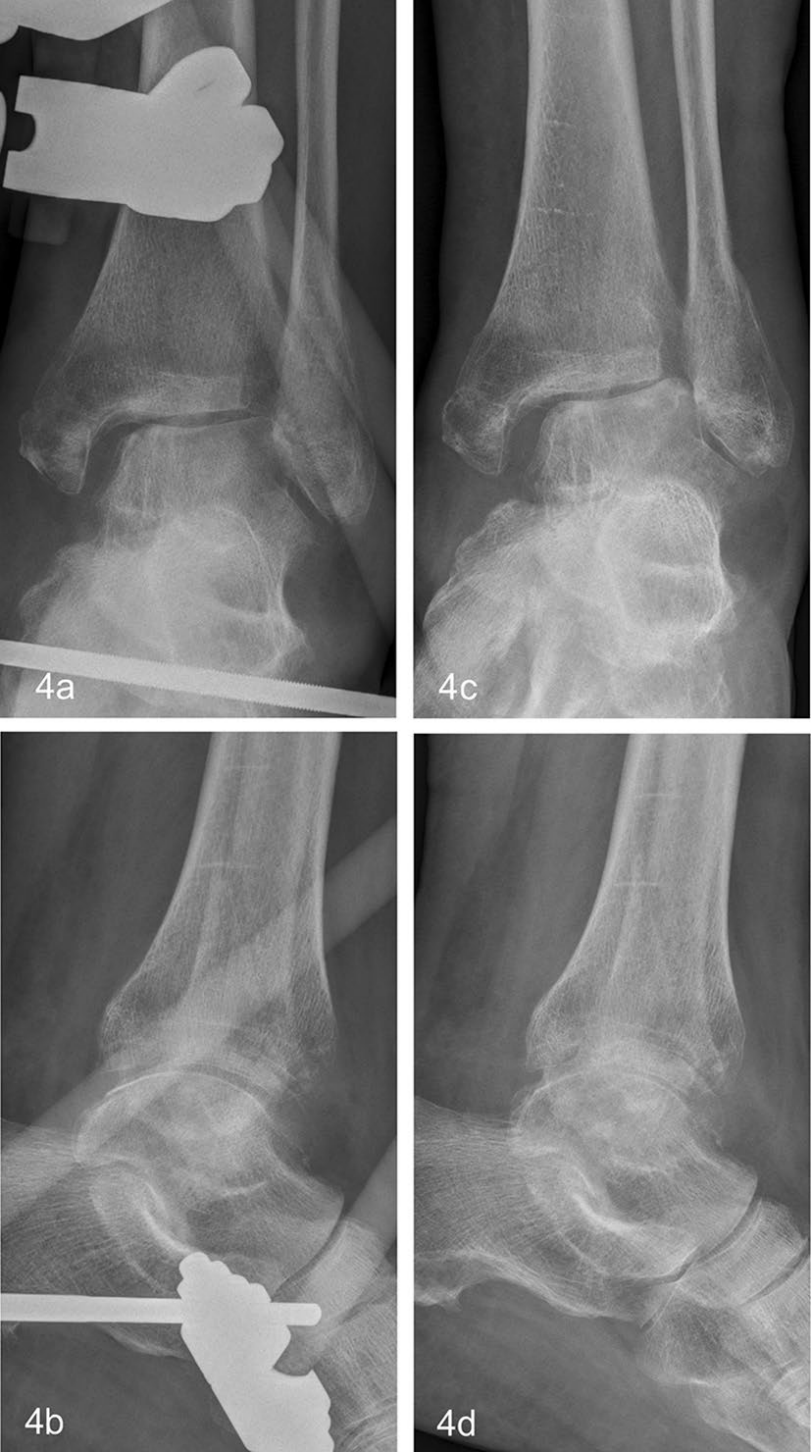
Same patient as in Fig. 3. Definite treatment with AEF was indicated due to the lack of soft tissue healing. **a**, **c** show lateral subluxation 60 days into therapy. Posttraumatic arthrosis with lateral incongruity was established 90 days after trauma and AEF removal after fracture healing (**b**, **d**)

Short-term radiographic outcomes were assessed using the stored X-ray (antero-posterior and lateral radiographs) images obtained immediately after EF removal. The radiological result was assessed as good if the joint congruity and the medial clear space were preserved and fracture healed without a step > 2 mm (Fig. 2c, d) [17]. Radiological results showing a widening of a medial clear space, incongruity of the ankle joint or a non-union with dislocation > 2 mm at the time of EF removal were assessed as poor (Figs. 3a, b and 4a–d).

Statistical analysis was performed with SPSS (version 24, Chicago, Illinois, USA), quantitative variables were presented as means and standard deviation (SD), maximum, minimum, and medians. Chi-square tests were used for categorical data and unpaired t-tests for continuous data. A *p* value < 0.05 was considered to be significant.

## Results

### Study groups

**Fig. 5:**
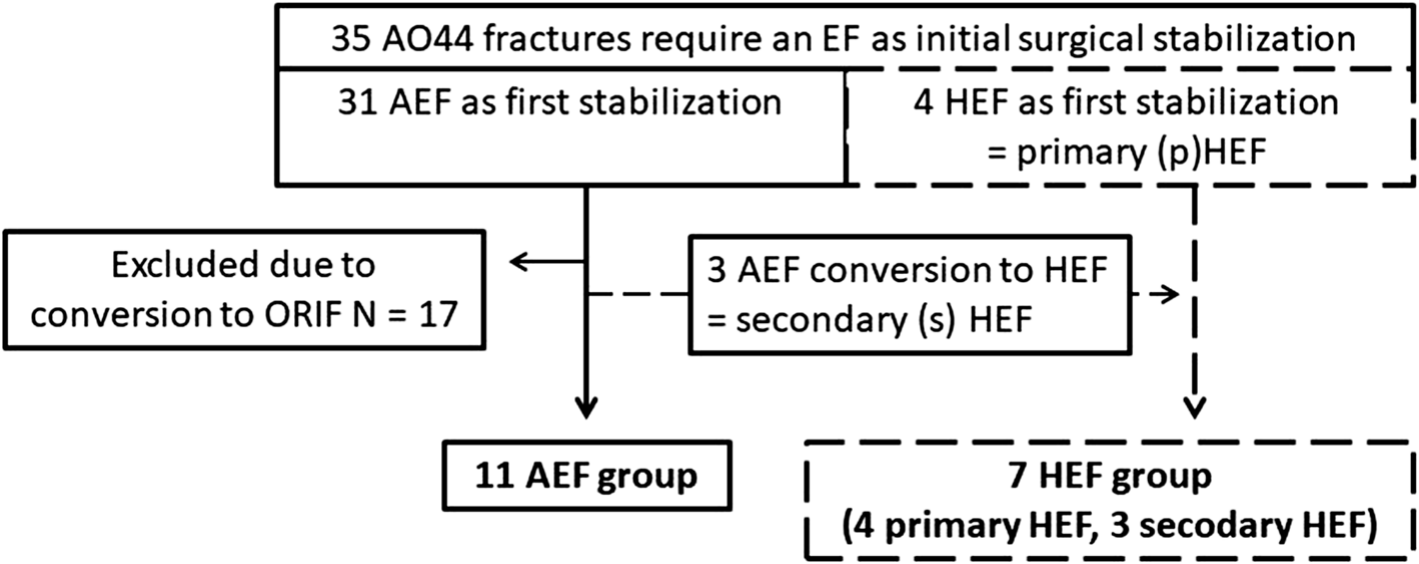
Patients flowchart and study groups, EF: external fixator, AEF: AO triangular external fixator, HEF: hybrid AO triangular external fixator, ORIF: open reduction and internal fixation

35 patients over 65 years of age were stabilized with AEF as the first surgical treatment for an AO 44 fracture. After adequate soft-tissue regeneration, 17 patients could be converted to ORIF. For 18 patients (3 men; 15 women; mean age 74 years; SD 6.2), the decision for finale treatment with an AEF was made. The decision was made in all patients due to poor soft tissue conditions with expected risk for wound complications and additional skin wounds in 4 cases, in 2 cases II° open fracture at medial malleolus and in one case each a chronic lymphedema or Charcot arthropathy (Table 1). Of these 14 were mounted as an AEF and 4 primarily as an HEF (pHEF group). After the decision for the final treatment with an AEF, three of the 14 patients who were initially stabilized with AEF were converted into an HEF by additional osteosynthesis in a second operation due to joint incongruity (secondary sHEF group; Figs. 1c, d and 2a, b). Two patients were converted to an external Ilizarov ring fixator. The patients’ flowchart is illustrated in Fig. 5.

**Table 1:** Patients demographic characteristics, complications, and radiological short-term outcome

	Total (*n* = 18)	AEF (*n* = 11)	HEF (*n* = 7)	*p*-value
Gender				
Female:male	15:3	10:1	5:2	1.000^c^
Mean age (years) (SD)	74 (6.1)	75 (6.7)	74 (6.1)	1.000^a^
Preoperative ASA (*n*; %)				
ASA I	2	2	0	1.000^b^
ASA II	6	3	3	
ASA III	10	6	4	
Preoperative living situation				
Self-employed	16	10	6	1.000^c^
Nursing home	2	1	1	
Mechanism of trauma				
Low-energy	16	10	6	1.000^c^
High-energy	2	1	1	
AO classification				
44 B2.2	9	4	5	1.000^c^
44 B3.2	8	6	2	
44 C	1	1		
Indication for continuation of therapy with EF				
Missing recovery of the critical soft swelling	10	7	3	
A skin wound	4	1	3	
II° open fracture	2	1	1	
Chronic lymphedema	1	1		
Charcot arthropathy	1	1		
Day of EF application after trauma (SD)	1.4 (0.9)	0.7 (0.6)	2.6 (0.7)	0.018^a^
Duration of EF therapy in days (SD)	74 (28)	77 (34)	70 (19)	0.536^a^
Complications requiring surgery (N)	4	3	1	1.000^c^
Radiographic outcome (N)				
Good	11	4	7	≤ 0.034^d^
Poor	7	5	0	

^a^*U*-test Exakte sig; ^b^Fisher’s test for ASA ≤ 2 vs. ASA > 2; ^c^Fisher’s test; ^d^Fisher’s test for AEF vs HEF until fracture healed

The AEF was combined with various osteosyntheses adapted to the soft tissue situation and fracture morphology, resulting in a heterogeneous pattern within the HEF group (Table 2).

**Table 2:** Overview of the used osteosynthesis in addition to AO triangular external fixator within the hybrid AO triangular external fixator (HEF) group (*n* = 7)

Patients (*n*)	Medial malleolar osteosynthesis	Lateral malleolar osteosynthesis	Posterior malleolar osteosynthesis
3		Intramedullary elastic nail	
1	Screws	Intramedullary elastic nail	
1	Screws		
1	Kirschner-wires	Plate ORIF	
1		Plate ORIF	Anterior- to-posterior screws

ORIF, open reduction and internal fixation

### Patients’ baseline characteristics

The age distribution of patients treated with an AEF (75 years, SD 6,7) or HEF (74 years, SD 6,1) until fracture had healed did not show any difference (*p* 1.000). Most patients (56%) were preoperatively assessed as ASA 3 without differences between the EF groups (*p *= 1.00; Table 1). According to the medical records, 16 (89%) patients lived as self-employed and 2 (11%) in a nursing home at the time before hospitalization.

The most frequent fracture types were the AO 44 B2 with 9 (50%) patients and the AO 44 B3 with 8 (44%, Table 1). Most fractures (16, 89%) were caused by low-energy trauma, while two fractures were caused by high-energy trauma.

### Course of the therapy

The fixator was applied on average 1.4 days (SD 0.9) after the accident, whereas it was applied later in the pHEF group (2.9 days; SD 0.7; p = 0.018). The average duration of EF therapy was 74 days (range 43–155; SD 28). There was no difference between HEF (70 days; SD 19) and AEF (77 days; SD 34; *p *= 0.536) or between the pHEF group (74 days; SD 24) and the sHEF group (65 days; SD 14; *p *= 0.400).

In 4 cases (22%), a complication requiring surgical revision occurred, which was equally distributed between the study groups. Three patients suffered a secondary dislocation during AEF therapy. Of these, two patients were converted to an external Ilizarov ring fixator 11 days, respectively, 15 days after initiation of AEF therapy. One patient required closed reduction and additional K-wire stabilization 24 days after the start of AEF therapy. One patient of the HEF group suffered a pin tract infection with need to HEF removal on day 83. In one patient, a loosening of the external fixator without surgical consequences was documented. A detailed overview of the treated patients is given in Table 3.

**Table 3:** Main characteristics of the included patients: 1 poor soft tissue conditions

Sex (age in years)	Fracture type	ASA	Indication for initial stabilization with AEF	Decided fixator type	Complications	Surgical revision (day after beginning of AEF therapy)	Rad. outcome/D of EF therapy (days)
Male (69)	B2.2	3	1	AEF			Poor—lat subl/62
Female (70)	B2.2	2	1 + lat subl in cast	AEF	Loos of AEF, no surg nec		Poor—lateral sub-luxation/60
Male (81)	B3.2	2	1 + lat subl in cast	AEF			Poor—lat subl/71
Female (88)	B3.2	2	1 + lat subl in cast	AEF			Good/43
Female (71)	B2.2	3	1 + wound at upper ant leg	AEF			Good/65
Female (72)	C2.3	3	1 + II° open frac med	AEF			Good/155
Female (76)	B3.2	2	1 + chronic lymphedema	AEF			Good/92
Female (68)	B3.2	3	1	AEF			Poor—lat subl/92
Female (79)	B3.2	2	1 + lat subl in cast	AEF	Pin inf. + lat disl > 2 mm in AEF	Calcaneotalotib K-wire stab (24)	Poor—lat subl/50
Female (68)	B3.2	3	1 + charcot arthropathy	AEF	Lat disl > 2 mm in AEF	Conv to ring fix (11)	Poor/120
Female (72)	B2.2	3	1 + lat subl in cast	AEF	Frac MT I + V, loos of AEF	Ring fix calcaneotalotib K-wire stab (15)	Poor—lat subl/82
Female (74)	B2.2	2	1 + wound at upper ant leg	HEF (AEF + fib e-nail)			Good/56
Male (74)	B2.2	3	1	HEF (AEF + med. screw OS)	Pininf + bleeding at pin	Removal of the HEF (83)	Good/81
Female (68)	B2.2	2	1 + wound at upper ant leg	HEF (AEF + fib e-nail + med. scew OS)			Good/59
Male (71)	B2.2	3	1 + lat subl in cast	HEF (AEF + fib e-nail)			Good/99
Female (81)	B2.2	3	1 + wound at ant upper leg	HEF (AEF + fib e-nail)			Good/52
Female (82)	B3.3	3	1 + lat subl in cast	HEF (AEF + fib plate OS + med K-wire)			Good/89
Female (66)	B3.3	2	1 + II° open fracture med	HEF (AEF + fib plate IS + post scews OS)			Good/56

AEF, AO trinangular external fixator; HEF, hybrid AO external fixator; rad., radiological; du, duration; loos, loosening; surg, surgery; nec, necessary; rec, recovery; ant, anterior; pin, pintract; inf., infection; lat, lateral; sublux, subluxation; disl, disclocation; calcaneotalotib, calcaneotalotibial; stab., stabilization; fib, fibular; e-nail, elastic nail; conv, conversion; fix, fixator; med., medial; OS, osteosynthesis; post, posterior; frac., fracture; MT, metatarsale

### Short-term radiographic and clinical outcome

The radiographic outcome of the patients in whom the decision for a definite treatment with AEF were made, was considered good in 11 (69%) patients and poor in 7 (31%) patients.

Good results at the time of EF removal were significantly more often in the HEF group (*n* = 7; 100%) than in the AEF group, regardless of whether treatment in this fixator was continued until fracture healing occurred (*n* = 4; 36%; *p *= 0.018). This result was also confirmed when only those patients treated were considered who were treated with HEF or AEF until fracture healing (7 [100%] vs. 4 [44%] out of 9; *p *= 0.034, Table 1). The two patients of the AEF group who were converted to a ring fixator showed insufficient radiological short-term outcome (Table 3).

## Discussion

Insufficient soft tissue recovery of the vulnerable skin of geriatric polymorbid patients despite external fixation is associated with an increased risk of complications. Therefore, techniques deviating from the standard may be considered for the definite treatment [9]. The present study describes the radiological outcome of ankle fractures in geriatric patients treated with AO fixation (AEF) until fracture healing and especially investigated the effect of additive osteosynthesis in addition to AEF in sense of hybrid external fixator (HEF).

It could be shown that the short-term radiographic results after therapy with a hybrid external fixator (HEF) were better than after AEF therapy alone (*p *< 0.05). The ankle joint of all patients treated with HEF could be healed in anatomical position. The duration of EF therapy was highly variable with a range of 43–155 days, but comparable in both groups (HEF 70 days; SD 19 and AEF 77 days; SD 34; *p *> 0.05).

The majority (56%) of patients analyzed were preoperatively assessed as ASA 3. As in other studies investigating alternative, less commonly used stabilization methods, most patients were polymorbid (ASA 3), which is associated with an increased complication rate after ORIF [6, 8, 12]. It was not possible to retrospectively determine the extent to which internal comorbidities influenced the decision for the final EF therapy, but it confirms that this patient population requires particular awareness. Corresponding to the literature, local risk factors such as missing recovery of soft tissue, vulnerable skin or surgical site wounds were the main reasons for the decision again secondary ORIF [9, 11].

There is consensus that gross dislocated fractures should be immediately reduced and retained. In these situations, external fixation, like an AEF, is an option until definitive internal fixation becomes feasible. In a comparable patient population, Meijer et al. [12] described that the radiographs showed suboptimal anatomy in 67% after Steinmann pin and 50% after prolonged EF therapy (32 days). These results were confirmed in the present study after AEF stabilization alone. But it could be demonstrated that significantly better radiological short-term results were achieved after HEF stabilization compared to the therapy with AEF alone. The goal of the initial EF is still the restoration and stabilization of the anatomy in order to achieve the best possible soft tissue healing and good conditions for a secondary ORIF, which represents the current gold standard. But the results support that already during initial fracture stabilization with an AEF, the option of prolonged or final treatment with an EF should be considered in polymorbid geriatric patients [11, 12, 18]. Depending on the soft tissue situation and the fracture morphology, additional less invasive osteosyntheses should be considered [7, 19, 20]. There is no uniform approach to additive procedures either in the current literature or in our institution, which reflects a limitation of the present study. On the one hand, additional procedures may be temporary and will be removed during conversion to ORIF or at the time of EF removal after fracture healing, e.g., temporary retrograde calcaneotalotibial K-wire stabilization. It could be shown that temporary retrograde calcaneotalotibial K-wire stabilization is not an obvious trigger for osteoarthritis in this patient group [19, 20]. Without additional external stabilization, good radiological results are only achieved in 33% [12]. On the other hand, procedures which are also used in the context of a definitive ORIF, e.g., percutaneous screw osteosynthesis of the medial or posterior malleolus could be considered. A single retrograde intramedullary screw fixation (self-tapping 4.2 mm screws or 4.5 mm cortical screws) in unstable distal fibula fractures in patients aged 65 years and older achieved good radiographic results in only 52% [21, 22]. Results after stabilization of the fibula with elastic nails alone in this patient group are not available. This represents the procedure most often added to the AEF in this study and, in our opinion, is not an obstacle to later conversion to ORIF. It can be used in sense of a modified Koval technique during a conversion to ORIF [23]. Zwipp and Amlang [18] recommended that in certain situations an applied external fixator be left in place despite conversion to ORIF until wound healing is complete to reduce complications. Two patients in the study were successfully treated with an additive AEF in addition to ORIF without infection occurring during treatment (Table 3).

The distal fibula nail and tibiotalocalcaneal (TTC) nail arthrodesis can be considered as alternatives to the HEF procedure presented but are not generally available. A randomized study could show that wound infections are less frequent after fibula nail than after ORIF of unstable ankle fractures in an elderly population [24]. However, wounds around the nail entry points are relative contraindications. The data available for TTC nail are encouraging, but controversial [25]. Therefore, according to the literature the TTC nail should only be reserved for polymorbid patients with very low demands [26].

Although the minimally invasive technique of HEF stabilization in poor soft tissue offers advantages, therapy with the fixator over several weeks (70 day) is a challenge for geriatric patients and is comparable to the Ilizarov fixator, which represents an alternative type of EF [11]. Over this time, consequent care of the EF is required to prevent pin tract infections [11]. In addition to the size and weight of the EF, compliance with a recommended partial weight bearing can be difficult for geriatric patients, which is difficult to control. At least for ORIF, it has been shown that a partial or no weight bearing is associated with a low rate of wound and bony complications [27].

### Limitations

The study has several limitations due to its retrospective design and the high age of the patients. As the patients were not available for follow-up visits, mainly due to their age, neither long-term clinical nor radiological results can be discussed. Therefore, the endpoint was defined as the radiological outcome (mortise and lateral view) immediately after EF removal at time of the fracture had healed, which was an acceptable limitation for the authors. To what extent the partial load was respected by the patients and what effect it has on the outcome of both groups cannot be answered by the study. But both groups were comparable in terms of ASA classification and age. Due to the rarity of the ankle fractures definitely treated in the AEF, the study groups were small and heterogeneous in terms of surgical management within the HEF group. Nevertheless, a significant difference in the short-term radiographic outcomes at the time of fracture healing between the two stabilization methods could be demonstrated.

### Future directions

Based on the presented data, it would be desirable to establish a standardized procedure in the sense of a HEF if an initial EF stabilization for soft tissue recovery is necessary in this specific patient group within a prospective study design. Another interesting point would be to analyze the impact of the HEF technique described in this study on soft tissue recovery time and complication rate after secondary ORIF in all age groups that initially require EF stabilization.

### Conclusion

In this study, closed or limited open fixation in addition to an AO fixator (AEF) in ankle fractures with critical soft tissue showed better short-term radiological results after fracture healing. Thus, according to these data, if an AEF is considered as prolonged or final therapy for an ankle fracture with soft tissue problems, it should be done or completed as a HEF. Additional closed maneuvers should be performed depending on the fracture morphology and soft tissue conditions. These can be performed as part of the initial stabilization or secondary in the case of insufficient soft tissue healing.

## Data Availability

The datasets used and/or analyzed during the current study are available from the corresponding author on reasonable request.
